# LncRNAs in serum-derived extracellular vesicles are potential biomarker and correlated with immune infiltration in gastric cancer

**DOI:** 10.3389/fimmu.2025.1533111

**Published:** 2025-01-24

**Authors:** Juan Ding, Yunyan Teng, Rongshu Cui, Jin Liu, Ke Xiao, Zhaogang Dong, Yi Zhang, Xiaofei Xu

**Affiliations:** ^1^ Department of Clinical Laboratory, Qilu Hospital of Shandong University, Jinan, China; ^2^ Center for Reproductive Medicine, Department of Obstetrics and Gynecology, Qilu Hospital of Shandong University, Jinan, China

**Keywords:** gastric cancer, extracellular vesicles, long non-coding RNA, diagnosis, prognosis

## Abstract

**Background:**

Long non-coding RNAs (lncRNAs) in extracellular vesicles (EVs) have been confirmed as effective non-invasive biomarkers for multiple diseases. However, their expression and clinical value in gastric cancer (GC) remain poorly understood.

**Materials and methods:**

Serum EV RNA was extracted from four patients with GC and four healthy controls, followed by high-throughput RNA sequencing. LncRNAs were further validated in training and validation sets using quantitative real-time reverse transcription polymerase chain reaction.

**Results:**

A total of 37,684 lncRNAs were obtained, and 10 lncRNAs were selected based on the criteria (*P* < 0.05 and |log_2_FoldChange| ≥1). Serum EV lncRNA RMRP, RPPH1, and linc-ROR were significantly higher in patients with GC than in those with chronic gastritis, atypical hyperplasia, or healthy control (all *P* < 0.05). Three lncRNAs were also significantly correlated with tumor diameter, lymphatic metastasis, distal metastasis, and TNM stage (all *P* < 0.05). The area under the curve (AUC) values for lncRNA RMRP, RPPH1, and linc-ROR were 0.727, 0.774, and 0.811, respectively. Corresponding sensitivity and specificity were 63.4% and 85.4%, 50.7% and 89.6%, and 78.5% and 66.7%. The combination of these three lncRNAs with carcinoembryonic antigen (CEA) yielded an AUC of 0.909, with a sensitivity and specificity of 83.3% each. Furthermore, high EV linc-ROR and RMRP expression levels were associated with worse disease-free survival and overall survival (OS). Univariate and multivariate Cox regression analyses confirmed that linc-ROR was the only independent prognostic factor for GC. Finally, the lncRNA-miRNA-mRNA network showed that three lncRNAs were predicted to interact with 15 miRNAs and 69 mRNAs. In addition, lncRNA RMRP and linc-ROR were correlated with immune cell infiltration, including neutrophils, central memory CD4 T cells, macrophage, and natural kill T cells.

**Conclusion:**

EV lncRNAs are prospective biomarker and correlated with immune cell infiltration in GC. It provides a foundation for the development of serum EV-targeted novel biomarkers and immunotherapy targets of GC.

## 1 Introduction

Gastric cancer (GC) is a significant contributor to the global burden of cancer and is one of the most common and deadly malignancies globally (1). It is estimated that there were 1 million new cases of GC and 769,000 deaths in 2020 (2). Therefore, the insufficiency in early detection and treatment is the main reason of high mortality (3). Currently, endoscopy and pathological biopsy are the gold standards that have increased the number of treatable cancers. However, these approaches are invasive, costly, and time-consuming (3, 4). As for blood markers, they are useful in GC monitoring. Traditional GC detection of tumor markers includes carcinoembryonic antigen (CEA), carbohydrate antigen 19-9 (CA19-9), and carbohydrate antigen 72-4 (CA72-4), but they all lack sensitivity and specificity (5). For example, our previous research has found that the expression of CA19-9 and CA72-4 shows no difference between groups (6). So, the diagnostic and prognostic biomarker with higher sensitivity and specificity is few in GC. Actually, therapeutic strategies play a crucial role in survival of GC. The primary treatment approach has surgical resection followed by adjuvant chemotherapy and radiotherapy. However, its clinical benefits are usually limited. The targeted therapy and immunotherapy are novel strategies and have demonstrated remarkable efficacy in improving the survival rate of GC patients. Anti-PD-1/PD-L1, immune checkpoint inhibitors, is an important advancement in this field (7). CD8+T and NK cells are complementary cytotoxic effectors and have been actively explored for cancer immunotherapy (8). Tumor infiltrating immune cells are a key component of tumor microenvironment (TME) as promising therapeutic efficacy indicators (9). The higher level of infiltration typically correlates with better clinical outcome. Understanding the role of immune cell infiltration and its related factors will facilitate the diagnosis and therapy of GC. Therefore, there is an urgent need for identifying novel biomarkers and exploring its correlation with immune infiltration.

Long non-coding RNAs (lncRNAs) are RNA molecules that have a transcript length exceeding 200 nucleotides and do not possess protein-coding ability. They regulate gene expression through interactions with protein, RNA, and DNA (10). LncRNAs, which were once regarded as transcriptional noise, have now emerged as crucial regulators of gene expression and important actors in cancer biology (11). For example, lncRNA-CDC6 promotes breast cancer progression and functions as a ceRNA to target CDC6 by sponging microRNA-215 (12). Similarly, lncRNA also plays an important role in the development of GC. LncRNA affects GC-related carcinogenic signaling cascades including pathways for PI3K/AKT/mTOR, p53, Wnt/β-catenin, JAK/STAT, and others (13). Moreover, Li et al. report that H19 may regulate the immune cell infiltration in carcinogenesis of GC through miR-378a-5p/SERPINH1 signaling, such as B cells, CD4+ T cells (14). The upregulation of OTX2-AS1 is associated with immune-infiltrating cells including Th17 cells, NK cells, and may be a useful biomarker for immunotherapy outcome of stomach adenocarcinoma (15). In addition, multiple studies have found that lncRNA can be used as biomarkers for the diagnosis and prognosis of cancer. In breast cancer, a study found that lncRNA CBR3-AS1 can be used as a biomarker with high sensitivity (16). In GC, studies have also demonstrated the feasibility and importance of lncRNAs as biomarkers, such as lncRNA PTCSC3 (17), lncRNA CADM1-AS1 (18), and others.

Accumulating studies have shown that lncRNAs are encapsulated by extracellular vesicles (EVs), and can stably exist in peripheral blood, which makes them novel non-invasive tumor marker (19). EVs are produced by many cells and secreted into the extracellular environment, serving as intercellular communication media. The contents contained in EVs, including lipids, proteins, mRNA, and lncRNA, can reflect the changes in various cellular pathophysiological states (20). The expression pattern of lncRNAs is wide in various type of cancer. The stability in circulating body fluids is helpful for diagnosing and monitoring tumors (21). For example, three lncRNAs (AC015922.2, AL135905.1, and LINC00921) are enriched in bile EVs and may be potential markers for tumors such as cholangiocarcinoma (22). In breast cancer, it is reported that five EV lncRNAs have been identified including C15orf54, AL157935.1, LINC01117, SNHG3, and AL355974.2, and these lncRNAs can serve as diagnostic and prognostic biomarkers (23). It is worth noting that high LINC00996 expression exhibits strong correlation with immune cell infiltration including NK cells and dendritic cells, suggesting potential benefits from immunotherapy (24). However, knowledge on EV lncRNAs in GC is still limited. Their value as biomarkers and correlation with immune infiltration needs further investigation.

In the current study, sequencing and qRT-PCR were employed to identify novel EV lncRNAs. After screening and validation, three EV lncRNAs were observed to be aberrantly expressed in GC, including the RNA component of mitochondrial RNA processing endoribonuclease (RMRP), Homo sapiens ribonuclease P RNA component H1 (RPPH1), and long-intergenic non-protein coding RNA, regulator of reprogramming (linc-ROR). They were also significantly correlated with tumor diameter, metastasis, and TNM stage. Moreover, these EV lncRNAs had good clinical value for GC diagnosis and prognosis. LncRNA RMRP and linc-ROR were correlated with immune cell infiltration, including neutrophils, central memory CD4 T cells, macrophage, and natural kill T cells.

## 2 Materials and methods

### 2.1 Patients and control subjects

Serum samples from four patients with GC and four healthy controls from Qilu Hospital of Shandong University, collected between December 2016 and December 2017, were analyzed to screen differentially expressed EV lncRNAs using high-throughput sequencing. To validate these lncRNAs, quantitative real-time RT-PCR (qRT-PCR) was conducted on a training set comprising 14 healthy controls and 28 patients with GC. Subsequently, the sample size was 48 healthy controls and 144 patients with GC as the validation set. Data on demographic and clinicopathological variables, such as age, gender, and hypertension, were recorded. Given the complex progression of GC from a normal condition to chronic gastritis, atypical hyperplasia, and finally GC, these lncRNAs were also analyzed in 48 patients with chronic gastritis and 43 patients with atypical hyperplasia. GC staging was performed according to the Union for International Cancer Control/American Joint Committee on Cancer TNM staging system (eighth edition). Inclusion and exclusion criteria were consistent with those in our previous study (25). Ethical approval of this study was granted by the Ethics Committee on Scientific Research of Shandong University Qilu Hospital (approval no. KYLL-2015-097).

### 2.2 Sample collection

Five milliliters of venous blood were collected from each participant using a vacuum tube (SST^TM^ II, BD-Belliver Industrial Estate, Plymouth, UK). Participants were instructed to abstain from eating or drinking prior to blood collection. Blood samples were left at room temperature for 1h to coagulate fully, centrifuged at 3000×g for 10 min to separate the serum, then aliquoted and stored at −80°C.

### 2.3 High-throughput sequencing

EVs were isolated using exoEasyMaxi kit (cat. no. 76064; Qiagen GmbH), and RNA was extracted according to operating procedures described in our previous study (26). Ribo-Zero rRNA Removal Kits (Illumina, San Diego, CA, USA) were employed to remove rRNAs from total RNA. A sequencing library was constructed using the TruSeq Stranded Total RNA Library Prep Kit, with quality control and quantification were performed on a BioAnalyzer 2100 instrument (Agilent Technologies, Santa Clara, CA, USA). Following to Illumina sequencing instructions, a 10 pM library was denatured into single-stranded DNA molecules, captured on an Illumina flow cell, and amplified *in situ* into clusters. Finally, sequencing was conducted in paired-end mode (PE mode) for 150 cycles using an Illumina HiSeq sequencer.

### 2.4 Sequencing data analysis

Sequencing was performed on an Illumina HiSeq 4000 sequencer to obtain paired-end reads. Quality control was conducted using Q30. The Cutadapt software (v1.9.3) was employed to remove 3’ adaptor sequences and low-quality reads yielding high-quality reads. Hisat2 software (v2.0.4) (https://daehwankimlab.github.io/hisat2/) was used to align these high-quality reads to the human reference genome (UCSC HG19). Following this, the guidance of the Ensembl GFT gene annotation file was used with Cuffdiff software (part of the Cufflinks suite) to obtain transcript-level lncRNA and mRNA FPKM (fragments per kilobase of exon per million fragments mapped) values, representing the expression profiles of lncRNAs and mRNAs. Fold change and *P* values were calculated between the two groups to screen differentially expressed lncRNAs and mRNAs. LncRNA target genes were predicted based on their proximity to nearby genes. The RNA sequencing dataset (GSE165394) has been uploaded to the Gene Expression Omnibus database.

### 2.5 lncRNA-miRNA-mRNA network analysis

The construction of network was derived from the correlation among mRNA, miRNA, and lncRNA. The different lncRNA–miRNA interactions were predicted through TargetScan and miRANDA, of which top miRNAs with the strongest blinding to each lncRNA, were selected. The target mRNAs of miRNAs were also predicted and then intersected with our sequencing data. The diagram of lncRNA-miRNA-mRNA network analysis was illustrated using cytoscape software.

### 2.6 Quantitative real-time RT-PCR

The expression of EV lncRNAs was verified using quantitative real-time RT-PCR. Serum EV RNA was extracted and reverse transcribed into cDNA using the all-in-one first-strand cDNA synthesis kit (Cat: QP006, GeneCopoeia Company, Rockville, MA). The cDNA was diluted at a 1:5 ratio. PCR amplification was carried out using a CFX96 system (BIO-RAD, USA) and All-in-One™ qPCR Mix (Cat: QP001, GeneCopoeia Company). The protocol was as follows: an initial denaturation of 95°C for 10 min, followed by 40 cycles at 95°C for 15 s, 62°C for 20 s, and 72°C for 10 s. Primer sequences were listed in **Table 1**. Using GAPDH as an internal reference, the relative expression levels of lncRNAs were calculated using the 2 ⁻ΔΔCT method.

**Table 1:** Primer sequences used for qRT-PCR analysis.

LncRNA	Primer type	Primer sequence	Amplicon length
RMRP	Forward	5'-GAGGACTCTGTTCCTCCCCT-3'	122 bp
	Reverse	5'-TACGCTTCTTGGCGGACTTT-3'	
RN7SL2	Forward	5'-GGACCACCAGGTTGCCTAAG-3'	137 bp
	Reverse	5'-GGTCTCGCTATGTTGCTCAGGC-3'	
RPPH1	Forward	5'-GGTGAGTTCCCAGAGAACGG-3'	160 bp
	Reverse	5'-GGTACCTCACCTCAGCCATT-3'	
CTD-2184D3.5	Forward	5'-CTGGCTGCGATGTGGTAACT-3'	138 bp
	Reverse	5'-CCAGACTCTTAACGGCTTGT-3'	
Linc-ROR	Forward	5'-AGTTATAGTTCTTCCAGGTCTCAGG-3'	218 bp
	Reverse	5'-GGTTCTAAGCAGAGTGGCGA-3'	
ZNRF3-IT1	Forward	5'-GATTGGAGACAGAGAAACTGCT-3'	133 bp
	Reverse	5'-CTTCCTCCTCTTCTCTCACTA-3'	
RP11-431K24.1	Forward	5'-CCACAAGTCGTGTGTTTCCC-3'	129 bp
	Reverse	5'-ACGGTTTTGCTCTCGACTCT-3'	
RP11-32B5.7	Forward	5'-GCATGGCACACAATCTCTAGC-3'	140 bp
	Reverse	5'-GCTCCTCTTCCTCTGGTCGA-3'	
RN7SL4P	Forward	5'-GATCGCTTGAGCCCAGGAGT-3'	120 bp
	Reverse	5'-CTCCTTAGGCAACCTGGTGGT-3'	
MALAT1	Forward	5'-GACTTCAGGTCTGTCTGTTCT-3'	135 bp
	Reverse	5'-CAACAATCACTACTCCAAGC-3'	

### 2.7 Fecal occult blood tests

The colloidal gold-based fecal occult blood diagnostic kit (Chemtron Biotech Co., Shanghai, China) was used to detect gastrointestinal bleeding in all participants.

### 2.8 White blood cell count and differential

One milliliter venous blood specimens were collected by BD vacutainer EDTA-K_2_ tubes. White blood cell count (WBC) was performed by Automated Hematology Analyzer XN-9000 (Sysmex Corporation, Kobe, Japan) according instrument operation. White cell differential (WDF) channel was used to differentiate neutrophil, monocyte, and lymphocyte, based on cell complexity (side-scattered fluorescent intensity), cell size (forward scattered light), and fluorescence signal (side fluorescent light). Neutrophil-to-lymphocyte ratio (NLR) and monocyte-to-lymphocyte ratio (MLR) were calculated.

### 2.9 Single sample gene set enrichment analysis and immune infiltration analysis

Transcriptome RNA sequencing data and their clinical parameters were downloaded in TPM (transcripts per million) format from TCGA-STAD project (https://portal.gdc.cancer.gov/). TPM values were transformed [log_2_(TPM+1)] to normalize data for further analysis. The gene of each sample was quantitatively analyzed by ssGAEA algorithm using “GSVA” package in R to estimate the abundance of immune infiltration based on immune cell subtypes. Furthermore, according to median value, lncRNAs (RMRP and linc-ROR) were divided into high expression and low expression. Wilcoxon rank sum test was used to compare the difference in immune cell infiltration, and Spearman correlation analysis was used to analyze the relationship between lncRNAs and immune infiltration. The correlation coefficient (R) and *P*-value were calculated.

### 2.10 Follow-up

The survival status of 144 patients with GC was monitored. The clinical endpoints of the analyses were disease-free survival (DFS) and overall survival (OS) over a 5-year period. DFS and OS were defined as the time between the date of surgery and the day of confirmed death, or recurrence, respectively. Survival, recurrence, and death data were obtained through telephone communications and recorded. The survival period was calculated accordingly.

### 2.11 Statistical analysis

The Kolmogorov-Smirnov test was performed to determine the distribution of data in each group. Data were presented as median and interquartile range. The levels of serum EV lncRNAs among different groups, including clinicopathological variables, were evaluated using the Mann-Whitney *U* test, Kruskal-Wallis test, or Spearman’s correlation analysis. Receiver operating characteristic (ROC) analysis was conducted to assess the diagnostic value of serum EV lncRNAs in GC. The area under the curve (AUC) was calculated to evaluate the clinical utility of lncRNAs. Cutoff values were determined using the Youden index (sensitivity + specificity -1). A *P* value of < 0.05 was considered statistically significant. In the 5-year follow-up data, univariate and multivariate Cox proportional hazards regression analyses were conducted to identify independent risk factors for DFS and OS in GC. The hazard ratio (HR) and 95% confidence interval (CI) were calculated. Kaplan–Meier analysis and the log-rank test were used to estimate GC survival curves. The R package "ggplot2" (version 3.3.6) was used to visualize the results. SPSS software (version 25.0) and MedCalc software (version 8.0) were used for statistical analyses.

## 3 Results

### 3.1 Characteristics of serum EVs lncRNAs sequencing

**Figure 1:**
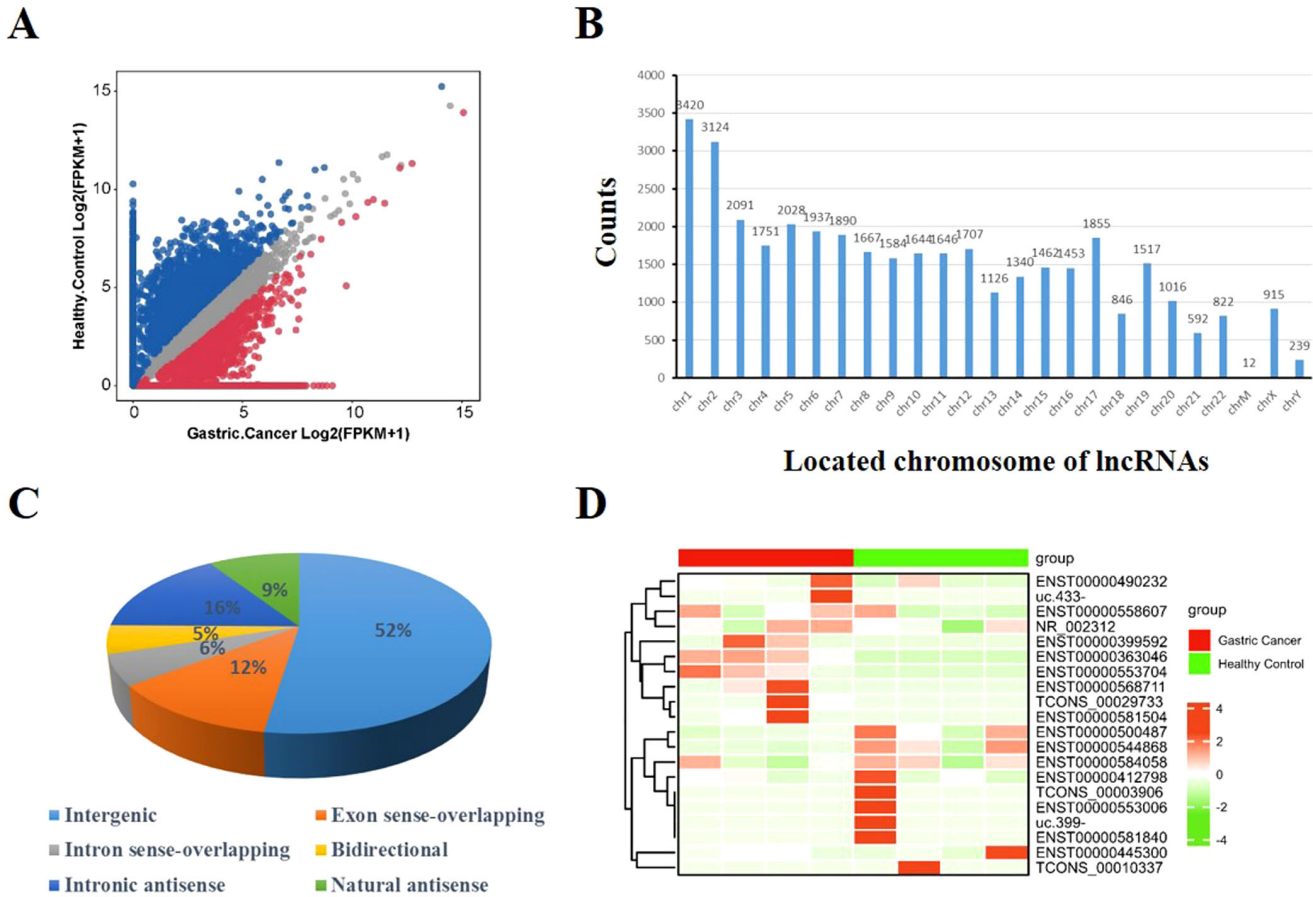
Characteristics of EV lncRNAs identified by sequencing. **(A)** Scatter plot illustrated the expression of lncRNAs [log_2_(FPKM+1)]. **(B)** The location distribution of lncRNAs on chromosomes 1 to 22, and chrM, chrX, chrY. *Y*-axis represented counts of lncRNAs in chromosome. **(C)** The proportion in percentage of six categorizations of lncRNA: intergenic, exon sense-overlapping, intron sense-overlapping, bidirectional, intronic antisense, natural antisense. **(D)** Heatmap represented top 10 upregulated and top 10 downregulated lncRNAs in gastric cancer compared with healthy controls.

A total of 37,684 lncRNAs in serum EVs was detected in control group and GC group, and visualized by scatterplot (**Figure 1A**). LncRNAs were located on all chromosomes (**Figure 1B**). According to the location of their parent genes, lncRNAs were divided into six categories, including intergenic, exon sense-overlapping, intron sense-overlapping, bidirectional, intronic antisense, natural antisense for 52%, 12%, 6%, 5%, 16%, 9%, respectively (**Figure 1C**). Furthermore, 61 different lncRNAs were selected, of which 29 lncRNAs were significantly upregulated, 32 lncRNAs were downregulated according to *P* < 0.05 and |log2FoldChange| ≥ 1. Top 10 of upregulated and downregulated lncRNAs were visualized in **Figure 1D**.

### 3.2 Validation of EV lncRNAs expression using qRT-PCR in the training set

**Figure 2:**
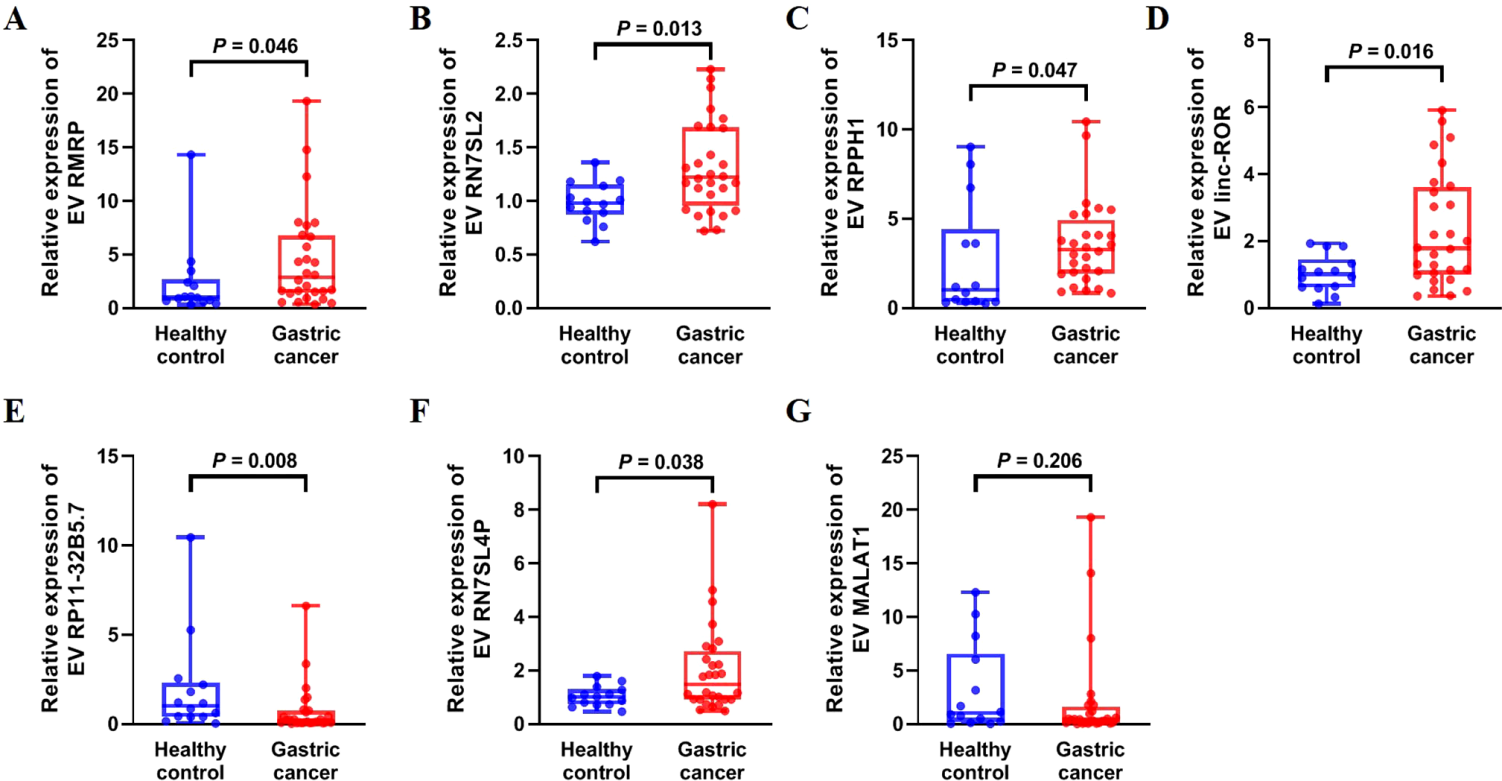
Expression of upregulated and downregulated serum EV lncRNAs in 14 healthy controls and 28 patients with gastric cancer in the training set. **(A)** RMRP, **(B)** RN7SL2, **(C)** RPPH1, **(D)** linc-ROR, **(E)** RP11-32B5.7, **(F)** RN7SL4P, and **(G)** MALAT1. The statistical method used between two groups was Mann-Whitney *U* test. Each plot represented the expression level of lncRNA in each patient.

Ten lncRNAs were selected for validation by qRT-PCR, including five upregulated lncRNAs (RMRP, RN7SL2, RPPH1, CTD-2184D3.5, and linc-ROR) and five downregulated lncRNAs (ZNRF3-IT1, RP11-431K24.1, RP11-32B5.7, RN7SL4P, and MALAT1) in serum EVs from patients with GC compared with healthy controls. The results showed that the expression levels of serum EV lncRNAs RMRP, RN7SL2, RPPH1, and linc-ROR were significantly higher in GC than in healthy controls (**Figures 2A–D**, all *P* < 0.05). LncRNA RP11-32B5.7 was downregulated in GC (**Figure 2E**, *P* < 0.05), and these trends were consistent with the sequencing results. Although serum EVs lncRNA RN7SL4P was upregulated in GC compared with the healthy control group, this finding was inconsistent with the sequencing result (**Figure 2F**). There was no significant difference in the expression of serum EV lncRNA MALAT1 between the two groups (**Figure 2G**, *P* > 0.05). Additionally, serum EVs lncRNAs CTD-2184D3.5, ZNRF3-IT1, and RP11-431K24.1 could not be amplified due to their extremely low-expression levels. Therefore, five lncRNAs (RMRP, RN7SL2, RPPH1, linc-ROR, and RP11-32B5.7) were selected for further experiments.

### 3.3 Validation of EVs lncRNAs expression in the validation set

**Figure 3:**
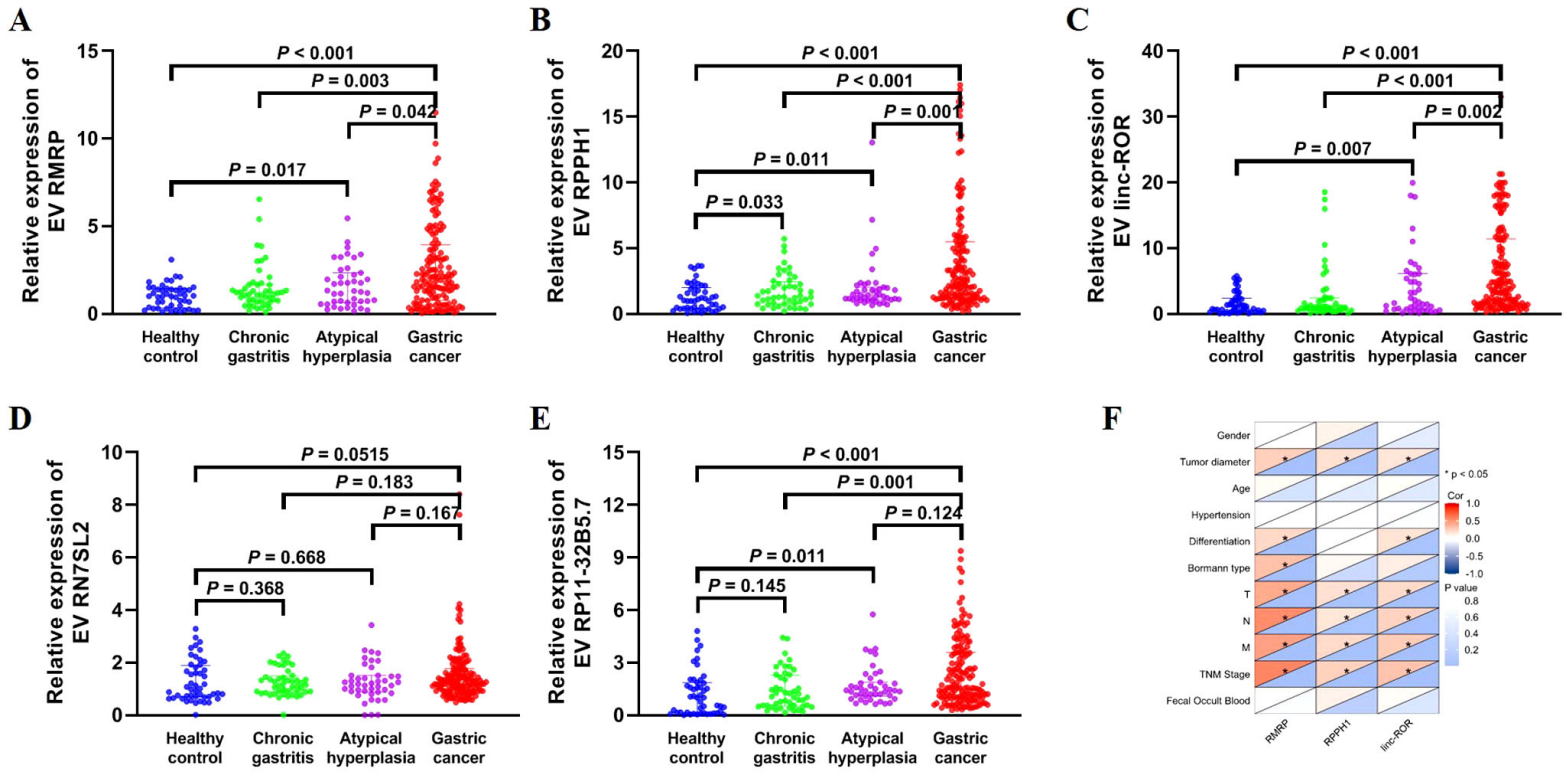
Expression of EV lncRNAs across four groups: 48 healthy control, 48 chronic gastritis, 43 atypical hyperplasia and 144 gastric cancers in the validation set. **(A)** RMRP, **(B)** RPPH1, **(C)** linc-ROR, **(D)** RN7SL2, and **(E)** RP11-32B5.7. The statistical method used between two groups was Mann-Whitney *U* test. **(F)** Heatmap of Spearman correlation analysis showing the relationship between EV lncRNAs and clinicopathological variables of gastric cancer. T, invasion depth. N, lymphatic metastasis. M, distal metastasis. *: *P* < 0.05.

We further confirmed the expression of the five EV lncRNAs in the validation set. **Figure 3A** demonstrated that the expression of serum EV lncRNA RMRP in patients with GC (1.965 [0.765–3.945]) was significantly higher than that in healthy controls (1.000 [0.325–1.435]), chronic gastritis (1.160 [0.705–1.670]), and atypical hyperplasia (1.300 [0.640–2.340]) (all *P* < 0.05). Meanwhile, the expression of lncRNA RMRP in atypical hyperplasia was higher than that in healthy controls (*P* < 0.05). Similarly, the expression of EV RPPH1 in GC (2.555 [1.235–5.498]) was obviously higher than that in healthy controls (1.000 [0.395–1.995]), chronic gastritis (1.330 [0.762–2.445]), and atypical hyperplasia (1.430 [1.070–2.020]) (all *P* < 0.05). LncRNA RPPH1 expression in chronic gastritis and atypical hyperplasia was higher than that in healthy controls (all *P* < 0.05, **Figure 3B**). Serum EV linc-ROR in GC (4.290 [1.613–11.41]) was higher than that in healthy control (1.000 [0.375–2.388]), chronic gastritis (1.000 [0.5725–2.418]), and atypical hyperplasia (1.790 [0.690–6.110]) (all *P* < 0.05, **Figure 3C**). It was also higher in atypical hyperplasia than in healthy controls (*P* < 0.05). However, no significant difference was observed in the expression of lncRNA RN7SL2 among the four groups (**Figure 3D**, *P* > 0.05). The trend for lncRNA RP11-32B5.7 was inconsistent with the results from the training set (**Figure 3E**). Based on these findings, we further evaluated the clinical value of lncRNA RMRP, RPPH1, and linc-ROR.

### 3.4 Correlation between EV lncRNAs and clinicopathological variables in gastric cancer

The correlation between three lncRNAs and clinicopathological variables, including gender, age, TNM stage, and others, was further analyzed (**Figure 3F**; **Table 2**). The results showed that lncRNA RMRP, RPPH1, and linc-ROR were significantly correlated with tumor diameter, lymphatic metastasis, distal metastasis, and TNM stage (all *P* < 0.05). There was no significant relationship between the three lncRNAs and gender, age, hypertension, or fecal occult blood (all *P* > 0.05). LncRNA RMRP was associated with the Borrmann type (*P* = 0.001). A significant correlation was observed between invasion depth and lncRNA RPPH1 (*P* < 0.001) and linc-ROR (*P* = 0.035). These findings suggest that these lncRNAs may be involved in the development and progression of GC.

**Table 2:** Correlations between EV lncRNAs and clinicopathological variables [median (interquartile range)].

Variables	N	RMRP			RPPH1			Linc-ROR		
		Median (interquartile range)	U value	*P* value	Median (interquartile range)	U value	*P* value	Median (interquartile range)	U value	*P* value
Gender										
Male	109	2.10 (0.63, 3.65)	1871.5	0.867	2.51 (1.17, 5.09)	1626.5	0.191	4.78 (1.72, 11.69)	1762	0.498
Female	35	1.76 (0.92, 4.81)			2.69 (1.57, 6.26)			3.12 (1.44, 7.47)		
Age										
≤61	75	1.86 (0.78, 3.66)	2497.5	0.719	2.29 (1.23, 5.27)	2519.5	0.786	3.93 (1.53, 10.20)	2334	0.311
>61	69	2.10 (0.76, 4.62)			2.92 (1.25, 5.80)			4.96 (1.95, 12.07)		
Hypertension										
No	111	2.08 (0.78, 3.65)	1766	0.756	2.60 (1.25, 5.26)	1771.5	0.775	4.23 (1.72, 11.72)	1751.5	0.704
Yes	33	1.86 (0.76, 4.80)			2.51 (1.08, 5.57)			4.64 (1.44, 7.77)		
Tumor diameter										
≤3cm	60	1.27 (0.43, 2.61)	1732.5	0.001	2.00 (1.01, 4.63)	1941	0.019	3.28 (1.53, 7.18)	2016.5	0.041
>3cm	84	2.24 (1.53, 4.58)			3.02 (1.49, 5.86)			5.40 (1.79, 13.12)		
Differentiation										
Well	13	0.68 (0.50, 1.30)	4.243^a^	0.120	1.63 (0.82, 13.32)	0.921^a^	0.631	2.16 (1.15, 7.26)	4.705^a^	0.095
Moderately	32	1.85 (0.85, 2.68)			3.20 (1.52, 5.84)			2.59 (1.44, 11.57)		
Poorly	99	2.23 (0.99, 4.81)			2.51 (1.25, 5.27)			4.91 (1.95, 11.93)		
Bormann type										
I	21	1.64 (0.47, 3.03)	20.657^a^	0.000	2.05 (1.44, 9.14)	2.926^a^	0.403	2.81 (1.21, 7.00)	4.987^a^	0.173
II	62	1.38 (0.46, 3.06)			2.56 (1.11, 4.75)			4.22 (1.72, 11.45)		
III	34	2.07 (1.47, 3.04)			3.45 (1.25, 8.92)			4.71 (1.44, 7.47)		
IV	27	3.66 (2.10, 5.99)			3.01 (1.41, 5.57)			9.17 (2.06, 18.02)		
Invasion depth										
T1	33	0.92 (0.36, 1.56)	25.255^a^	0.000	2.05 (1.15, 3.79)	8.595^a^	0.035	2.61 (1.53, 7.09)	7.417^a^	0.060
T2	20	1.05 (0.38, 4.13)			1.31 (0.94, 3.33)			3.96 (2.01, 8.65)		
T3	28	1.91 (1.12, 3.05)			3.37 (1.24, 5.81)			4.30 (1.74, 7.18)		
T4	63	2.76 (1.76, 5.14)			3.01 (1.57, 6.26)			7.29 (1.72, 16.10)		
Lymphatic metastasis										
No	50	0.66 (0.25, 1.46)	822.5	0.000	2.00 (1.01, 3.79)	1858.5	0.039	2.88 (1.34, 4.96)	1632	0.003
Yes	94	2.60 (1.64, 4.98)			2.84 (1.41, 5.80)			6.13 (1.88,13.04)		
Distal metastasis										
No	107	1.51 (0.49, 2.71)	791.5	0.000	2.07 (1.12, 4.80)	1414.5	0.01	3.71 (1.52, 7.77)	1252	0.001
Yes	37	4.54 (2.23, 6.28)			3.37 (2.04, 6.84)			9.89 (2.47, 16.41)		
TNM stage										
I	39	0.68 (0.25, 1.43)	45.801^a^	0.000	1.95 (1.01, 3.31)	10.652^a^	0.014	3.12 (1.21, 4.96)	14.713^a^	0.002
II	34	1.80 (0.57, 3.03)			2.48 (0.87, 5.26)			3.56 (1.52, 10.20)		
III	39	2.56 (1.59, 4.73)			2.25 (1.29, 4.75)			4.81 (1.53, 15.49)		
IV	32	4.15 (2.17, 6.18)			4.29 (2.21, 7.61)			9.24 (2.91, 16.40)		
Fecal Occult Blood										
No	103	1.96 (0.92, 3.66)	2023.5	0.697	2.13 (1.20, 4.98)	1800.5	0.169	4.21 (1.72, 9.66)	1929	0.419
Yes	41	1.97 (0.61, 4.98)			3.37 (1.46, 6.00)			5.47 (1.27, 15.92)		

a Chi-Square.

### 3.5 Diagnostic value of EV lncRNAs in gastric cancer

**Figure 4:**
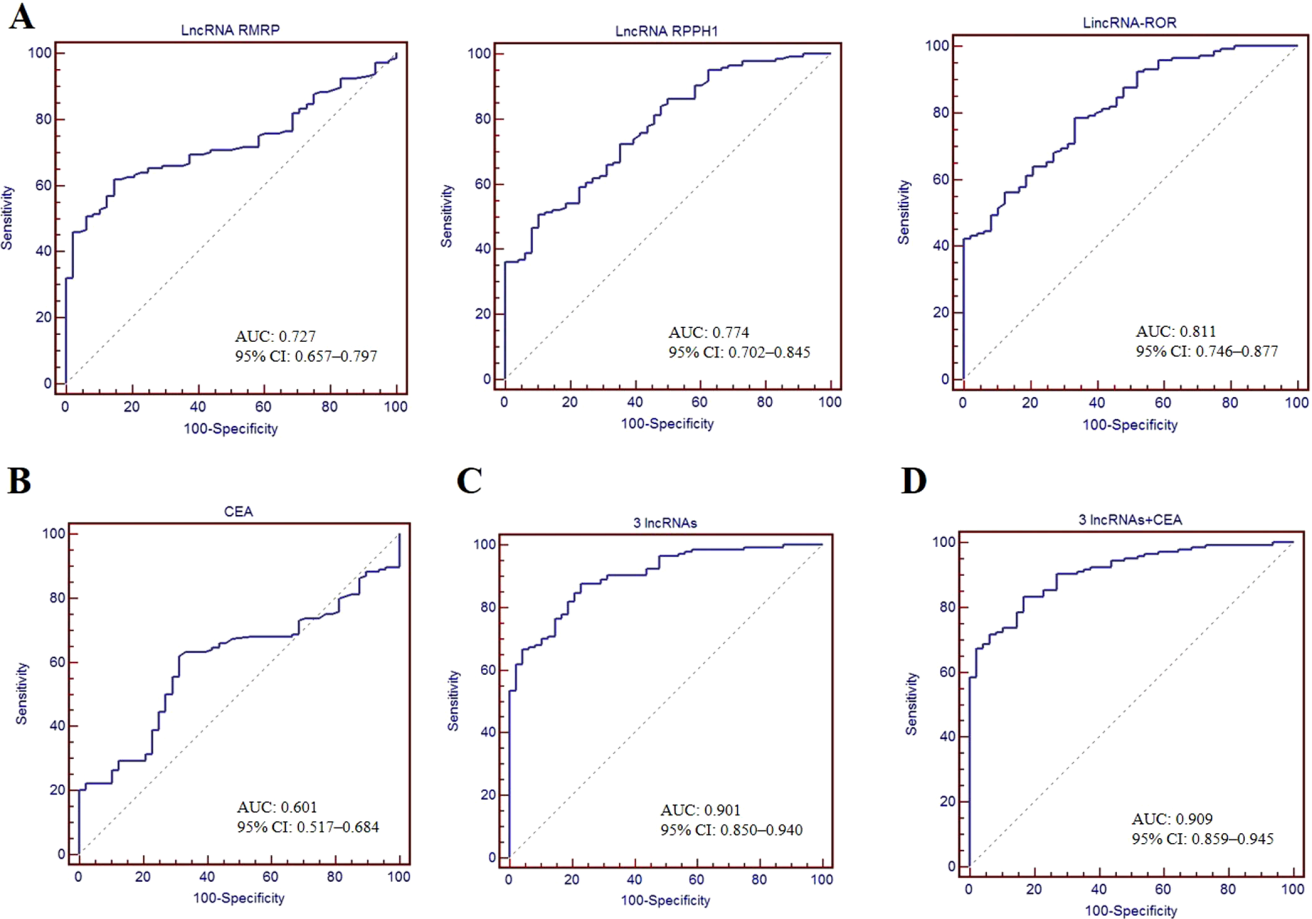
Diagnostic value of serum EV lncRNAs. ROC curves used to assess the diagnostic performance of lncRNA RMRP, RPPH1 and linc-ROR **(A)**, CEA **(B)**, combination of three lncRNAs **(C)**, and combination of three lncRNAs + CEA **(D)**. AUC with 95%CI was calculated.

To assess the diagnostic value of EV lncRNAs for GC, we constructed ROC curves. As shown in **Figures 4A, B**, the AUC of lncRNA RMRP (0.727 [95% CI: 0.657–0.797]), RPPH1 (0.774 [95% CI: 0.702–0.845]) and linc-ROR (0.811 [95% CI: 0.746–0.877]) were higher than that of CEA (0.601 [95% CI: 0.517–0.684]), demonstrating a high classification power for distinguishing patients with GC from healthy controls. The corresponding sensitivity and specificity were as follows: RMRP (63.4% and 85.4%), RPPH1 (50.7% and 89.6%), linc-ROR (78.5% and 66.7%), and CEA (61.8% and 68.7%), with cutoff values of 1.52, 2.47, 1.49, and 2.19 ng/ml, respectively. These findings suggest that EV lncRNAs may be more reliable markers than CEA for patients with GC. The combination of the three lncRNAs resulted in an AUC of 0.901 (95% CI: 0.850–0.940), with a sensitivity of 87.5% and a specificity of 77.1% (**Figure 4C**). Furthermore, combining these three lncRNAs with CEA, revealed an AUC of 0.909 (95% CI: 0.859–0.945), with sensitivity and specificity both at 83.3% (**Figure 4D**).

### 3.6 Survival analysis of EV lncRNAs in gastric cancer

**Figure 5:**
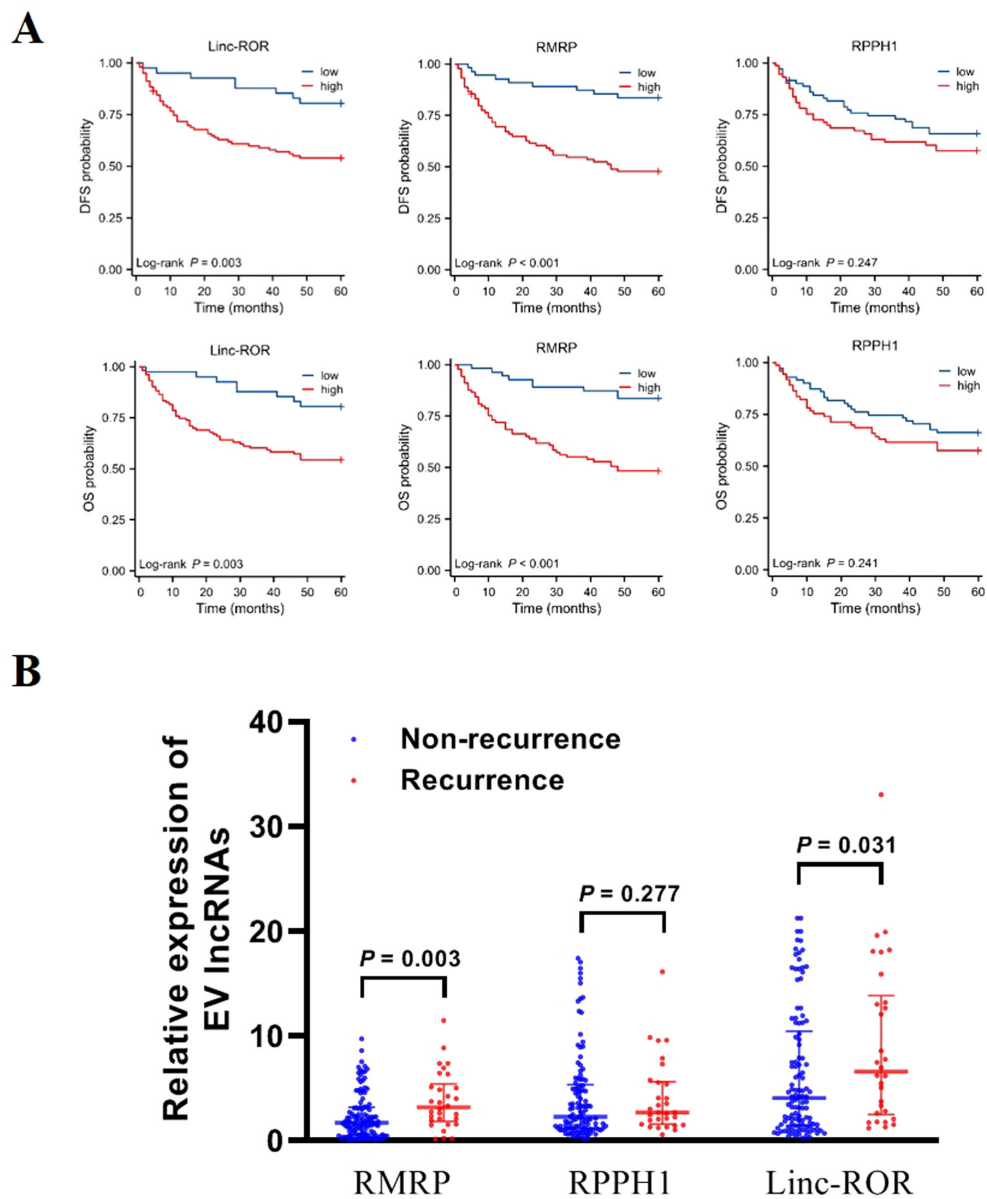
Prognosis value of EV lncRNAs in patients with GC. **(A)** Kaplan–Meier survival analysis for linc-ROR, RPPH1, and RMRP in relation to DFS and OS. **(B)** Expression of EV linc-ROR, RPPH1, and RMRP in patients with GC recurrence/non-recurrence. The statistical method used between two groups was Mann-Whitney *U* test.

We conducted follow-up assessments and categorized patients with GC into low and high EV lncRNAs groups (RMRP, RPPH1, and linc-ROR) based on the optimal cutoff values. Kaplan–Meier analysis demonstrated that patients with high levels of linc-ROR and RMRP had worse DFS (*P* = 0.003, *P* < 0.001, respectively) and OS (*P* = 0.003, *P* < 0.001, respectively). However, no difference was observed between lncRNA RPPH1 and DFS (*P* = 0.247) and OS (*P* = 0.241, **Figure 5A**). Univariate Cox regression analysis showed that DFS and OS were significantly correlated with several variables, including tumor diameter, differentiation, Bormann type, invasion depth, lymphatic metastasis, distal metastasis, TNM stage, and lncRNAs (RMRP and linc-ROR) (all *P* < 0.05, **Table 3**). These variables were then subjected to multivariate Cox regression analysis, which revealed that TNM stage (HR = 4.221, 95% CI = 1.271–14.023, *P* = 0.019) and high EVs linc-ROR (HR = 2.282, 95% CI = 1.020–5.106, *P* = 0.045) were independent prognostic factors for DFS. For OS, distal metastasis (HR = 1.886, 95% CI = 1.028–3.459, *P* = 0.040), TNM stage (HR = 3.939, 95% CI = 1.191–13.024, *P* = 0.025), and high EVs linc-ROR (HR = 2.326, 95% CI = 1.035–5.226, *P* = 0.041) were identified as independent prognostic factors (**Table 3**). Additionally, 30 (26.32%) of the144 patients with GC experienced recurrence within 5 years. RMRP and linc-ROR were associated with tumor recurrence (all *P* < 0.05, **Figure 5B**).

**Table 3:** Correlations between variables and survival outcomes (DFS and OS) in GC patients, determined using univariate and multivariate Cox regression analysis.

Variables		Total (N)	DFS				OS			
			univariate		multivariate		univariate		multivariate	
			HR (95% CI)	*P* value	HR (95% CI)	*P* value	HR (95% CI)	*P* value	HR (95% CI)	*P* value
Gender	Male	109	Reference				Reference			
	Female	35	0.767 (0.396-1.486)	0.432			0.766 (0.396-1.485)	0.430		
Age	≤61	75	Reference				Reference			
	>61	69	1.073 (0.632-1.821)	0.794			1.092 (0.644-1.853)	0.744		
Hypertension	No	111	Reference				Reference			
	Yes	33	0.737 (0.380-1.427)	0.365			0.745 (0.385-1.442)	0.382		
Tumor diameter	≤3cm	60	Reference		Reference		Reference		Reference	
	>3cm	84	3.462 (1.823-6.573)	< 0.001	0.818 (0.372-1.799)	0.617	3.376 (1.778-6.409)	< 0.001	0.775 (0.348-1.727)	0.533
Differentiation	Well+Moderately	45	Reference		Reference		Reference		Reference	
	Poorly	99	2.465 (1.242-4.894)	0.010	1.490 (0.728-3.049)	0.275	2.473 (1.246-4.909)	0.010	1.537 (0.752-3.142)	0.238
Bormann type	I+II	83	Reference		Reference		Reference		Reference	
	III+IV	61	4.028 (2.287-7.095)	< 0.001	1.669 (0.884-3.153)	0.114	3.927 (2.230-6.916)	< 0.001	1.603 (0.847-3.035)	0.147
Invasion depth	T1+T2	53	Reference		Reference		Reference		Reference	
	T3+T4	91	6.491 (2.776-15.177)	< 0.001	1.422 (0.437-4.633)	0.559	6.361 (2.721-14.871)	< 0.001	1.436 (0.435-4.739)	0.552
Lymphatic metastasis	No	50	Reference		Reference		Reference		Reference	
	Yes	94	9.668 (3.489-26.786)	< 0.001	1.761 (0.425-7.305)	0.436	9.546 (3.446-26.450)	< 0.001	1.815 (0.440-7.477)	0.410
Distal metastasis	No	107	Reference		Reference		Reference		Reference	
	Yes	37	5.469 (3.198-9.353)	< 0.001	1.690 (0.921-3.100)	0.090	5.684 (3.320-9.730)	< 0.001	1.886 (1.028-3.459)	0.040
TNM stage	I+II	73	Reference		Reference		Reference		Reference	
	III+IV	71	11.655 (5.252-25.864)	< 0.001	4.221 (1.271-14.023)	0.019	11.348 (5.115-25.175)	< 0.001	3.939 (1.191-13.024)	0.025
Fecal Occult Blood	No	103	Reference				Reference			
	Yes	41	1.054 (0.589-1.886)	0.860			1.063 (0.594-1.902)	0.837		
RMRP	low	55	Reference		Reference		Reference		Reference	
	high	89	4.182 (2.044-8.555)	< 0.001	0.897 (0.375-2.142)	0.806	4.196 (2.052-8.583)	< 0.001	0.903 (0.376-2.167)	0.819
RPPH1	low	71	Reference				Reference			
	high	73	1.369 (0.803-2.333)	0.248			1.375 (0.807-2.344)	0.241		
Linc-ROR	low	41	Reference		Reference		Reference		Reference	
	high	103	2.977 (1.406-6.307)	0.004	2.282 (1.020-5.106)	0.045	2.964 (1.400-6.278)	0.005	2.326 (1.035-5.226)	0.041

### 3.7 Construction of lncRNA-miRNA-mRNA network based on lncRNAs

**Figure 6:**
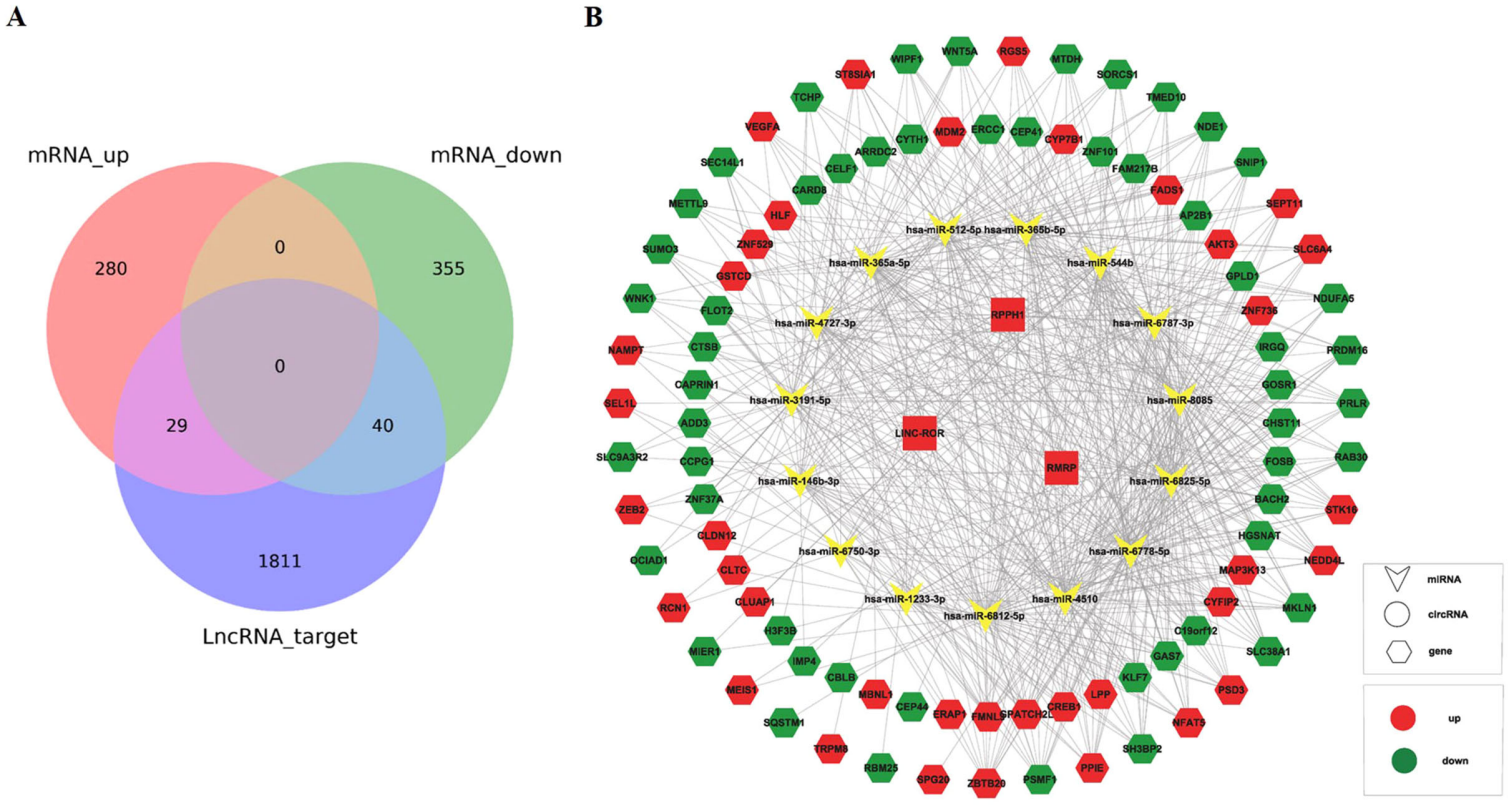
Construction of lncRNA-miRNA-mRNA network. **(A)** The Venn diagram showed the intersection of mRNAs in sequencing data and mRNAs from prediction by lncRNAs. **(B)** Construction of lncRNA-miRNA-mRNA network based on the selected lncRNA linc-ROR, RPPH1, and RMRP.

Base on the three upregulated lncRNAs, we constructed the network of lncRNA-miRNA-mRNA. The expression of mRNA in sequencing data had been listed in our previously published article (25), including 20,308 mRNAs (13,991 upregulated mRNAs and 6,317 downregulated mRNAs). The targeted miRNAs binding with three lncRNAs were predicted by TargetScan and miRNADA, of which top five miRNAs with the strongest blinding to each lncRNA were selected. The target mRNAs of 15 miRNAs (hsa-miR-146b-3p, hsa-miR-3191-5p, hsa-miR-6825-5p, hsa-miR-8085, hsa-miR-4510, hsa-miR-365a-5p, hsa-miR-365b-5p, hsa-miR-512-5p, hsa-miR-4727-3p, hsa-miR-6787-3p, hsa-miR-1233-3p, hsa-miR-6778-5p, hsa-miR-544b, hsa-miR-6812-5p, hsa-miR-6750-3p) were also predicted. Top 200 mRNAs with the highest binding affinity were used to intersect with mRNAs in our sequencing data, and 29 upregulated and 40 downregulated mRNAs were obtained (**Figure 6A**). Based on the above relationship, a network diagram of lncRNA-miRNA-mRNA were constructed (**Figure 6B**).

### 3.8 Correlation between lncRNAs and immune infiltration

**Figure 7:**
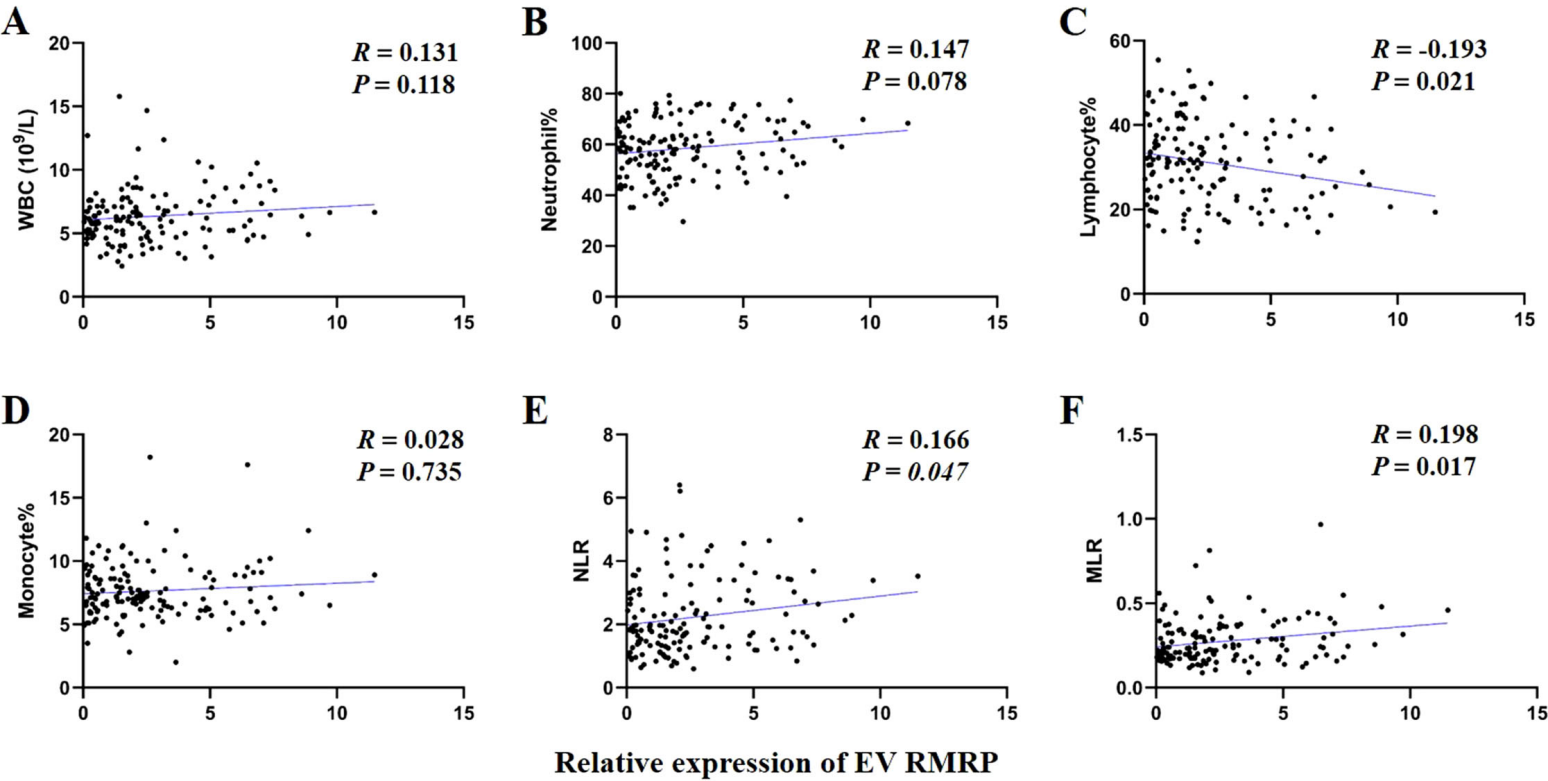
The correlation between EV lncRNA RMRP with white blood cell (WBC)–related markers. **(A)** WBC counts. **(B)** Neutrophil%. **(C)** Lymphocyte%. **(D)** Monocyte%. **(E)** Neutrophil-to-lymphocyte ratio, NLR. **(F)** Monocyte -to-lymphocyte ratio, MLR. The statistical method was Spearman correlation analysis. Neutrophil% was the percentage of neutrophils counts in WBC counts. Lymphocyte% was the percentage of lymphocytes counts in WBC counts. Monocyte% was the percentage of monocytes counts in WBC counts.

**Figure 8:**
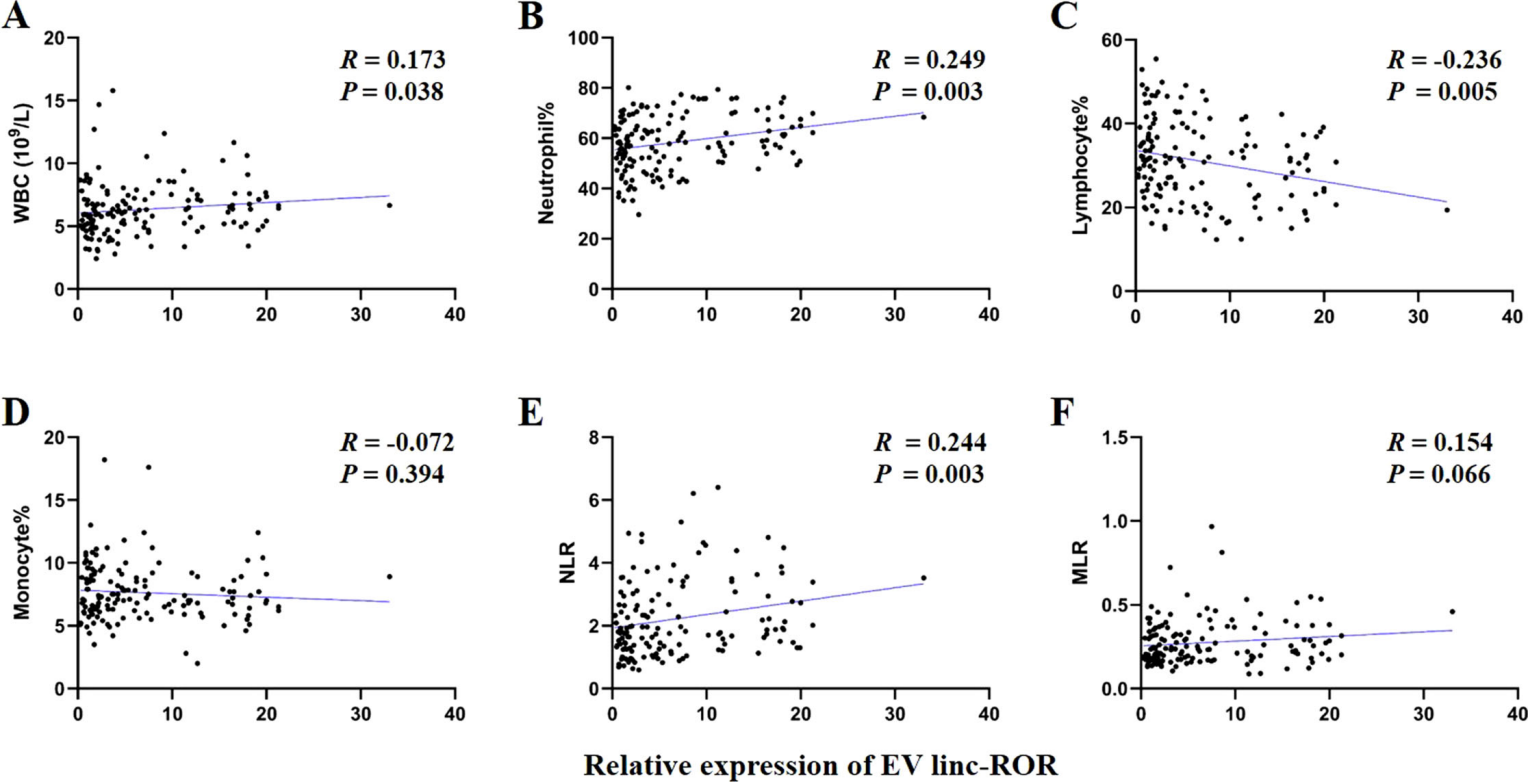
The correlation between EV linc-ROR with white blood cell (WBC)–related markers. **(A)** WBC counts. **(B)** Neutrophil%. **(C)** Lymphocyte%. **(D)** Monocyte%. **(E)** Neutrophil-to-lymphocyte ratio, NLR. **(F)** Monocyte-to-lymphocyte ratio, MLR. The statistical method was Spearman correlation analysis. Neutrophil% was the percentage of neutrophils counts in WBC counts. Lymphocyte% was the percentage of lymphocytes counts in WBC counts. Monocyte% was the percentage of monocytes counts in WBC counts.

**Figure 9:**
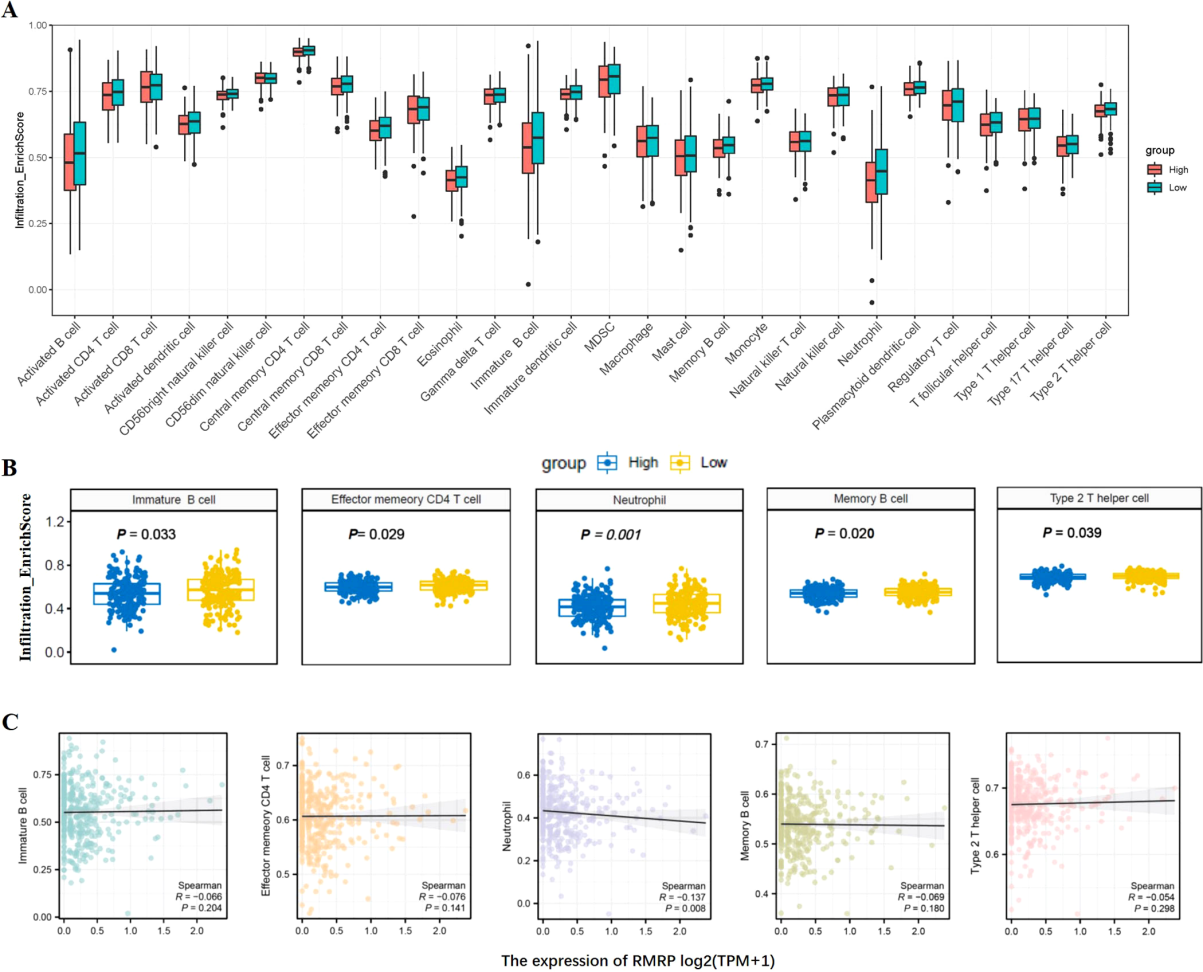
The landscape of immune cell infiltration and correlation analysis with lncRNA RMRP in STAD. **(A)** Box plots depicted immune cell infiltration differences between high and low lncRNA RMRP expression groups in the TCGA-STAD cohort. **(B)** The difference between five immune cells and with high/low lncRNA RMRP expression, including immature B cell, effector memory CD4 T cell, neutrophil, memory B cell, type 2 T helper cell. The statistical method was Mann-Whitney *U* test. **(C)** Spearman correlation analysis without RMRP grouping [log_2_(TPM+1)] was further shown in scatter plot.

**Figure 10:**
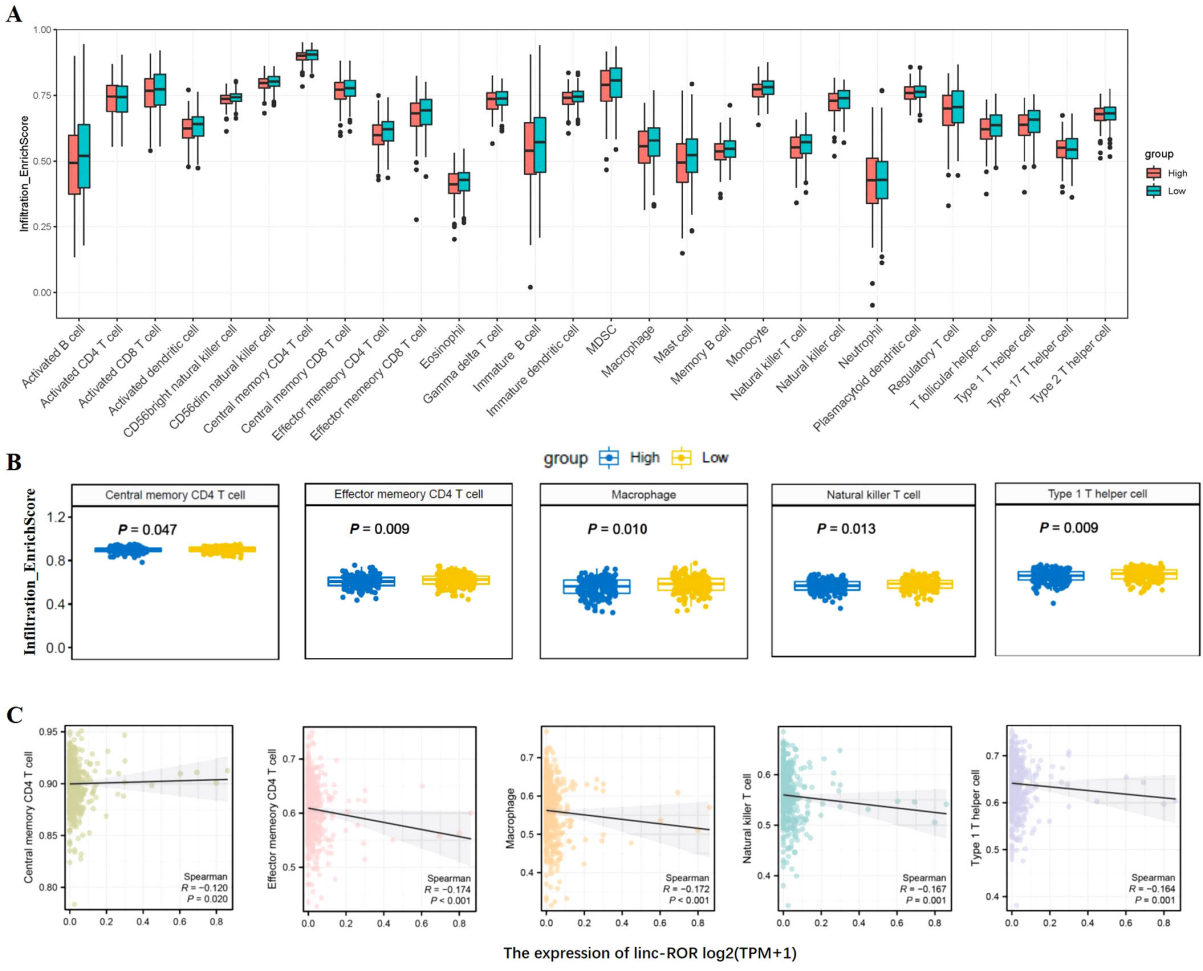
The landscape of immune cell infiltration and correlation analysis with linc-ROR in STAD. **(A)** Box plots depicted immune cell infiltration differences between high and low linc-ROR expression groups in the TCGA-STAD cohort. **(B)** The difference between five immune cells and with high/low linc-ROR expression, including central memory CD4 T cells, effector memory CD4 T cells, macrophage, natural kill T cells, and type 1 T helper cell. The statistical method was Mann-Whitney *U* test. **(C)** Spearman correlation analysis without linc-ROR grouping [log_2_(TPM+1)] was further shown in scatter plot.

The prognosis of GC is closely related with TME and immune therapy response. Immune cell infiltration in TME play a critical role in tumor development. The systemic inflammatory markers in serum can be used to predict outcome of cancer, for example, WBC counts, neutrophil, lymphocyte, monocyte, NLR, and MLR. Therefore, we first evaluated the correlation between lncRNAs and circulating WBC-related markers. Results showed that lncRNA RMRP was unrelated with WBC counts (**Figure 7A**), while positively related with neutrophil%, NLR and MLR in GC (**Figures 7B, E, F**). Linc-ROR was positively related with WBC counts, neutrophil% and NLR (**Figures 8A, B, E**), but unrelated with MLR (**Figure 8F**). Both of them were negatively related with lymphocyte%, while unrelated with monocyte% (**Figures 7C, D**, **8C, D**). To elucidate the potential role of lncRNAs in TME, the relationship between lncRNAs and immune infiltration cells was further confirmed. Results demonstrated that five immune infiltration cells were significantly related to high lncRNA RMRP expression, including immature B cell, effector memory CD4 T cell, neutrophil, memory B cell, type 2 T helper cell (**Figures 9A, B**). However, in the correlation analysis without grouping based on expression levels, lncRNA RMRP was found to be significantly correlated only with neutrophils (**Figure 9C**). Linc-ROR were significantly correlated with five immune infiltrating cells, including central memory CD4 T cells, effector memory CD4 T cells, macrophage, natural kill T cells, and type 1 T helper cell (**Figures 10A, B**). Notably, regardless of the expression grouping, linc-ROR exhibited significant associations with all five immune cell types (**Figure 10C**).

## 4 Discussion

GC is one of the most malignant tumors worldwide. Early diagnosis and treatment can significantly improve the survival rate of patients (1, 3). However, at present, there is a lack of effective biomarkers and therapy strategy, which severely affects the diagnosis and treatment of patients with GC. Immune therapy is a promising therapeutic efficacy, but there are still many patients cannot benefit from immunotherapy. In this study, we identified 37,684 lncRNAs using high-throughput sequencing, and subsequently verified 10 aberrantly expressed lncRNAs. The results showed that serum EV lncRNAs, specifically RMRP, RPPH1, and linc-ROR were upregulated in GC and were closely related to TNM stage, metastasis, and other clinicopathological factors. These lncRNAs may be involved in the carcinogenic process of GC. Furthermore, the combination of these three lncRNAs demonstrated good diagnostic value for GC. LncRNA RMRP and linc-ROR were also closely associated with the survival (DFS and OS) while linc-ROR serving as an independent prognostic factor for GC. In addition, lncRNA RMRP and linc-ROR were correlated with immune cell infiltration, including neutrophils, central memory CD4 T cells, macrophage, and natural kill T cells. These findings suggest that serum EVs lncRNAs could be reliable novel biomarkers and immunotherapy targets, contributing to improving diagnostic and therapeutic capabilities for GC.

EVs, including exosomes, play a crucial role in intercellular communication and are involved in disease progression of various diseases. LncRNAs are important components of EVs, and are abnormally expressed in various diseases, including cancer. Increasingly, studies have focused on the potential of lncRNAs as novel biomarkers. In this study, serum EV lncRNAs RMRP, RPPH1, and linc-ROR were upregulated in GC and exhibited good diagnostic value. These findings make EV lncRNAs promising candidates for the early screening and diagnosis of GC, which is consistent with previous studies (27). However, other lncRNAs were not selected for further analysis due to low expression or inconsistent trend between sequencing and qRT-PCR. The reason might be the different principles of two methods. qRT-PCR has a higher sensitivity and specificity by designing primer, as well as a low cost and simple operation, which is suitable for large-scale screening and validation of specific genes. While sequencing has stronger advantages in conducting whole genome or transcriptome analysis. High expression of lncRNA RMRP was found in plasma exosomes and was identified as a diagnostic biomarker for BLCA (28). However, another study reported that lncRNA RMRP was significantly downregulated in tissue specimens from patients with thyroid carcinoma (29). We speculate that this discrepancy may be due to the organ specificity of lncRNA RMRP, as well as the differences in the sample types used. This highlights the importance of considering expression differences when applying lncRNAs as diagnostic markers in future studies. LncRNA RPPH1 plays a role in pre-tRNA processing and associated with tumor progression (30). In colorectal cancer (CRC), RPPH1 was significantly upregulated in both tissue and exosomes, and was proposed as a potential therapeutic and diagnostic target (31), which aligns with our findings regarding its diagnostic potential. Linc-ROR, a 2604bp lncRNA located on 18q21.31, was initially identified in pluripotent and embryonic stem cells and has been shown to be dysregulated in patients with various disorders, including cancer (32). In this study, we observed that linc-ROR was upregulated in GC. Conversely, its expression was found to decrease in parathyroid tumors (33). Thus, previous studies, as well as our results, illustrate the diversity in the expression patterns of lncRNAs. As is well known, the traditional tumor markers often lack sufficient sensitivity and specificity. The combined detection of tumor biomarkers can improve both sensitivity and specificity (34). Our study confirmed this, as the AUC of the three lncRNAs was higher than that of CEA, and the combination of the three lncRNAs yielded an AUC of 0.901, with a sensitivity of 87.5% and a specificity of 77.1%. This combination may help distinguish patients with GC from healthy controls. Although these lncRNAs exhibited a good specificity for GC, their expression in other type cancers remained unclear. Further studies including more type cancers were guaranteed to validate specificity. Given these factors, producing a commercial reagent kit could be a good attempt, and might enhance the precision of GC diagnosis and treatment. Of course, before doing so, a larger size and multi-center studies should be conducted to further confirm these results.

Emerging evidence suggests that EV lncRNAs exert pivotal roles in tumor progression through various mechanisms. Our results showed that lncRNA RMRP, RPPH1, and linc-ROR were closely associated with size, metastasis and TNM stage, suggesting that they may be involved in the progression of GC. Recently, ceRNA regulatory network is reported to be related to the malignant biological behavior of cancer. For example, lncRNA RMRP can promote the proliferation and invasion of non-small cell lung cancer through miR-613/NFAT5 pathway (35). In GC, ceRNA mechanism of RMRP is also investigated, and RMRP/miR-206/cyclin D2 axis shows activity in regulating cell proliferation (36). Similarly, lncRNA RPPH1 and linc-ROR contribute to GC progression including proliferation, invasion, and migration by P21 (37) or miR-212-3p/FGF7 axis (38). Therefore, we also constructed ceRNA (lncRNA-miRNA-mRNA) network and predicted potential therapeutic target of GC. However, we do not carry out research about these mechanisms, and further research is guaranteed in future. Expanding our understanding of the function of EV lncRNAs will not only provide new insight into the pathogenic and chemo-drug resistance mechanisms of GC, but also pave the way for developing novel diagnosis and treatment options.

Currently, the prognosis of GC remains unsatisfactory. The main reason for this is the lack of reliable prognostic markers. Previous studies on EV lncRNAs as prognostic markers have also been reported (39). In our study, we analyzed the survival rates of three EV lncRNAs, combined with univariate and multivariate Cox regression analyses, and identified that RMRP and linc-ROR were associated with both DFS and OS in GC. However, lncRNA RPPH1 was not associated with prognosis. We speculate that this discrepancy may be due to the fact that different lncRNAs exert different functions, and RPPH1 has primarily been proposed as a diagnostic marker, rather than a therapeutic target. This observation prompted further analysis, which revealed that only linc-ROR was an independent prognostic risk factor for both DFS and OS in GC. This finding is consistent with previous studies showing that linc-ROR is a prognostic marker for renal cell carcinoma (40). Preliminary research, has explored the role of linc-ROR in modulating malignant phenotypes and remodeling the TME through signaling pathways such as RAS/RAF/MEK/ERK, WNT/β-catenin, and others (41). The transfer of lncRNAs via EVs may represent a new mechanism for understanding anti-cancer drug resistance. In circulation, EV linc-ROR may serve as a predictor of progression-free survival. Targeting lncRNAs, such as linc-ROR in pancreatic cancer and RMRP in bladder cancer, has led to a marked reduction in tumor volume and weight, as well as inhibited tumor metastasis in BALB/c nude mice or C57BL/6J male mice (42, 43). Based on previous studies and our results, it can be concluded that targeting lncRNAs is indeed an effective therapeutic strategy. Additionally, we find that both RMRP and linc-ROR are associated with GC recurrence, which provides indirect evidence supporting the above conclusion. A potential opportunity and challenge for future research lies in exploring the use of EV lncRNAs biomarkers and therapeutic targets.

Infiltration immunocytes in TME play a crucial role in predicting prognosis and therapeutic reaction. Immunotherapy is a novel strategy for GC and has attracted widespread attention. LncRNAs have been implicated in facilitating the evasion of immune surveillance by tumor cells. In this study, high lncRNA RMRP expression was significantly correlated with immune cells, including B cell, effector memory CD4 T cell, neutrophil, memory B cell, and type 2 T helper cell. However, further analysis showed that it was only correlated with neutrophils (no grouping). We speculated the reason might be attributed to the strong involvement of RMRP in innate immunity, with minimal association to adaptive immunity. This was further supported by our results about circulating WBC-related markers analysis. Neutrophil extracellular traps (NETs) is extensive extracellular web-like structures produced and released by activated neutrophils, intricately associated with tumor response to immunotherapy and chemotherapy. Previous report showed that lncRNA NEAT1 was response to NET treatment, and then participated in the regulation of GC invasion (44). LncRNA RMRP might also be involved in GC progression with a similar mechanism, but further experiments are needed to validate this conclusion. For linc-ROR, five immune infiltrating cells were markedly associated, including central memory CD4 T cell, effector memory CD4 T cell, macrophage, natural kill T cell, and type 1 T helper cell. This gave us a hint that it was highly relevant to T cell immunity, which was consistent with previous study. For example, lncRNA H19 recruited tumor-associated macrophages, leading to T cell exhaustion and the remodeling of immune microenvironment in glioblastoma (45). In GC, linc-ROR may also play an effect through this pathway in immunomodulation, prompting us it is a promising immunotherapeutic target for enhancing the efficacy of GC immunotherapy. NLR has been recognized as a marker of systemic inflammation, and is great clinical interest due to its accessibility and the ease of circulating the ratio from patient’s blood routine test. It has emerged as a potential prognostic factor in various cancers (46). Our results showed that both lncRNA RMRP and linc-ROR were positively related with NLR, negatively related with lymphocyte while unrelated with monocyte. The combination of lncRNA and NLR has the best diagnostic value in infectious diseases, such as RP11-248E9.5 and NLR in pneumonia (47). However, no report has been found about the application of lncRNA and NLR in cancers until now. This revelation highlights the importance of exploring the interaction between lncRNAs and immune cells and provides new insights into developing more effective treatment options.

In summary, our findings show that serum EV lncRNA RMRP, RPPH1 and linc-ROR are abnormally expressed in GC and are significantly correlated with tumor size, stage, metastasis, and other clinicopathological factors. These lncRNAs demonstrate superior diagnostic and prognostic performance and could be proposed as potential biomarkers, with significant clinical application value. Meanwhile, lncRNA RMRP and linc-ROR were correlated with immune cell infiltration, including neutrophils, central memory CD4 T cell, macrophage, natural kill T cell. However, there are certain limitations in our study. First, the sample size was small and there was a lack of external validation experiments. Therefore, the conclusions need to be further verified through multi-center and large-sample studies. Second, the current study did not investigate the underlying mechanisms, especially regarding their potential as immune therapeutic targets. Further research is required to determine the diagnostic and prognostic value of these lncRNAs and to elucidate the underlying mechanisms in GC.

## Data Availability

The datasets presented in this study can be found in online repositories. The names of the repository/repositories and accession number(s) can be found below: (GSE165394) had been uploaded in the Gene Expression Omnibus.

## References

[1] Puliga E Corso S Pietrantonio F Giordano S. Microsatellite instability in gastric cancer: between lights and shadows. Cancer Treat Rev. (2021) 95:102175. doi: 10.1016/j.ctrv.2021.102175 33721595

[2] Xia JY Aadam AA. Advances in screening and detection of gastric cancer. J Surg Oncol. (2022) 125:1104–9. doi: 10.1002/jso.26844 PMC9322671 35481909

[3] Matsuoka T Yashiro M. Novel biomarkers for early detection of gastric cancer. World J Gastroenterol. (2023) 29:2515–33. doi: 10.3748/wjg.v29.i17.2515 PMC10198055 37213407

[4] Moayyedi P Axon AT. Endoscopy and gastric ulcers. Endoscopy. (1995) 27:689–93. doi: 10.1055/s-2007-1005789 8903984

[5] Zhang R Chen X Chen G Zhao Z Wei Y Zhang F. Combined use of tumor markers in gastric cancer: A novel method with promising prognostic accuracy and practicality. Ann Surg Oncol. (2023) 30:8561–71. doi: 10.1245/s10434-023-14194-9 37718336

[6] Dong Z Sun X Xu J Han X Xing Z Wang D. Serum membrane type 1-matrix metalloproteinase (Mt1-mmp) mrna protected by exosomes as a potential biomarker for gastric cancer. Med Sci Monit. (2019) 25:7770–83. doi: 10.12659/MSM.918486 PMC6820360 31619663

[7] Jin X Liu Z Yang D Yin K Chang X. Recent progress and future perspectives of immunotherapy in advanced gastric cancer. Front Immunol. (2022) 13:948647. doi: 10.3389/fimmu.2022.948647 35844558 PMC9284215

[8] Zhou X Yu J Cheng X Zhao B Manyam GC Zhang L. The deubiquitinase otub1 controls the activation of cd8(+) T cells and nk cells by regulating il-15-mediated priming. Nat Immunol. (2019) 20:879–89. doi: 10.1038/s41590-019-0405-2 PMC6588407 31182807

[9] Hinshaw DC Shevde LA. The tumor microenvironment innately modulates cancer progression. Cancer Res. (2019) 79:4557–66. doi: 10.1158/0008-5472.CAN-18-3962 PMC6744958 31350295

[10] Xing C Sun SG Yue ZQ Bai F. Role of lncrna lucat1 in cancer. BioMed Pharmacother. (2021) 134:111158. doi: 10.1016/j.biopha.2020.111158 33360049

[11] Zhang Y. Lncrna-encoded peptides in cancer. J Hematol Oncol. (2024) 17:66. doi: 10.1186/s13045-024-01591-0 39135098 PMC11320871

[12] Kong X Duan Y Sang Y Li Y Zhang H Liang Y. Lncrna-cdc6 promotes breast cancer progression and function as cerna to target cdc6 by sponging microrna-215. J Cell Physiol. (2019) 234:9105–17. doi: 10.1002/jcp.27587 30362551

[13] Elimam H Abdel Mageed SS Hatawsh A Moussa R Radwan AF Elfar N. Unraveling the influence of lncrna in gastric cancer pathogenesis: A comprehensive review focus on signaling pathways interplay. Med Oncol. (2024) 41:218. doi: 10.1007/s12032-024-02455-w 39103705

[14] Li J Han T Wang X Wang Y Chen X Chen W. H19 may regulate the immune cell infiltration in carcinogenesis of gastric cancer through mir-378a-5p/serpinh1 signaling. World J Surg Oncol. (2022) 20:295. doi: 10.1186/s12957-022-02760-6 36104825 PMC9472414

[15] Han QL Cui Z Wang Q Pang F Li D Wang D. Upregulation of otx2-as1 is associated with immune infiltration and predicts prognosis of gastric cancer. Technol Cancer Res Treat. (2023) 22:15330338231154091. doi: 10.1177/15330338231154091 36740995 PMC9905030

[16] Torkashvand S Basi A Ajdarkosh H Rakhshani N Nafisi N Mowla SJ. Long non-coding rnas expression in breast cancer: cbr3-as1 lncrna as a sensitive biomarker. Asian Pac J Cancer Prev. (2021) 22:2897–902. doi: 10.31557/APJCP.2021.22.9.2897 PMC8850887 34582659

[17] Zhang G Chi N Lu Q Zhu D Zhuang Y. Lncrna ptcsc3 is a biomarker for the treatment and prognosis of gastric cancer. Cancer Biother Radiopharm. (2020) 35:77–81. doi: 10.1089/cbr.2019.2991 31702383

[18] Shi XY Sun YZ Li M Li HY. Lncrna cadm1-as1 serves as a new prognostic biomarker for gastric cancer. Eur Rev Med Pharmacol Sci. (2019) 23:232–8. doi: 10.26355/eurrev_201908_18652 31389606

[19] Wu Y Wang Y Wei M Han X Xu T Cui M. Advances in the study of exosomal lncrnas in tumors and the selection of research methods. BioMed Pharmacother. (2020) 123:109716. doi: 10.1016/j.biopha.2019.109716 31896067

[20] Urabe F Kosaka N Ito K Kimura T Egawa S Ochiya T. Extracellular vesicles as biomarkers and therapeutic targets for cancer. Am J Physiol Cell Physiol. (2020) 318:C29–39. doi: 10.1152/ajpcell.00280.2019 31693397

[21] Bhan A Soleimani M Mandal SS. Long noncoding rna and cancer: A new paradigm. Cancer Res. (2017) 77:3965–81. doi: 10.1158/0008-5472.CAN-16-2634 PMC8330958 28701486

[22] Zong H Yu W Lai H Chen B Zhang H Zhao J. Extracellular vesicles long rna profiling identifies abundant mrna, circrna and lncrna in human bile as potential biomarkers for cancer diagnosis. Carcinogenesis. (2023) 44:671–81. doi: 10.1093/carcin/bgad063 37696683

[23] Wang H Shu L Niu N Zhao C Lu S Li Y. Novel lncrnas with diagnostic or prognostic value screened out from breast cancer via bioinformatics analyses. PeerJ. (2022) 10:e13641. doi: 10.7717/peerj.13641 35855425 PMC9288825

[24] Nan K Zhang M Geng Z Zhang Y Liu L Yang Z. Exploring unique extracellular vesicles associated signatures: prognostic insights, immune microenvironment dynamics, and therapeutic responses in pancreatic adenocarcinoma. Mediators Inflammation. (2024) 2024:2825971. doi: 10.1155/2024/2825971 PMC11366062 39220187

[25] Xiao K Li S Ding J Wang Z Wang D Cao X. Expression and clinical value of circrnas in serum extracellular vesicles for gastric cancer. Front Oncol. (2022) 12:962831. doi: 10.3389/fonc.2022.962831 36059681 PMC9428625

[26] Xiao K Dong Z Wang D Liu M Ding J Chen W. Clinical value of lncrna ccat1 in serum extracellular vesicles as a potential biomarker for gastric cancer. Oncol Lett. (2021) 21:447. doi: 10.3892/ol.2021.12708 33868485 PMC8045156

[27] Jiang C Zhang J Wang W Shan Z Sun F Tan Y. Extracellular vesicles in gastric cancer: role of exosomal lncrna and microrna as diagnostic and therapeutic targets. Front Physiol. (2023) 14:1158839. doi: 10.3389/fphys.2023.1158839 37664422 PMC10469264

[28] Gao Y Wang X Luo H Chen C Li J Sun R. Exosomal long non-coding ribonucleic acid ribonuclease component of mitochondrial ribonucleic acid processing endoribonuclease is defined as a potential non-invasive diagnostic biomarker for bladder cancer and facilitates tumorigenesis via the mir-206/G6pd axis. Cancers (Basel). (2023) 15:5305. doi: 10.3390/cancers15215305 37958478 PMC10649581

[29] Luzon-Toro B Villalba-Benito L Fernandez RM Torroglosa A Antinolo G Borrego S. Rmrp, rmst, ftx and ipw: novel potential long non-coding rnas in medullary thyroid cancer. Orphanet J Rare Dis. (2021) 16:4. doi: 10.1186/s13023-020-01665-5 33407723 PMC7789680

[30] Lin YH Chen CW Cheng HC Liu CJ Chung ST Hsieh MC. Inhibition of lncrna rpph1 activity decreases tumor proliferation and metastasis through down-regulation of inflammation-related oncogenes. Am J Transl Res. (2023) 15:6701–17. PMC10767529 38186977

[31] Liang ZX Liu HS Wang FW Xiong L Zhou C Hu T. Correction: lncrna rpph1 promotes colorectal cancer metastasis by interacting with tubb3 and by promoting exosomes-mediated macrophage M2 polarization. Cell Death Dis. (2020) 11:465. doi: 10.1038/s41419-020-2661-3 32546789 PMC7297724

[32] Ghafouri-Fard S Pourtavakoli A Hussen BM Taheri M Kiani A. A review on the importance of linc-ror in human disorders. Pathol Res Pract. (2023) 244:154420. doi: 10.1016/j.prp.2023.154420 36989849

[33] Yu Q Hardin H Chu YH Rehrauer W Lloyd RV. Parathyroid neoplasms: immunohistochemical characterization and long noncoding rna (Lncrna) expression. Endocr Pathol. (2019) 30:96–105. doi: 10.1007/s12022-019-9578-3 31119524

[34] Fu P Gong B Li H Luo Q Huang Z Shan R. Combined identification of three lncrnas in serum as effective diagnostic and prognostic biomarkers for hepatitis B virus-related hepatocellular carcinoma. Int J Cancer. (2022) 151:1824–34. doi: 10.1002/ijc.34201 35802466

[35] Yang M Ke H Zhou W. Lncrna rmrp promotes cell proliferation and invasion through mir-613/nfat5 axis in non-small cell lung cancer. Onco Targets Ther. (2020) 13:8941–50. doi: 10.2147/OTT.S255126 PMC7494237 32982286

[36] Shao Y Ye M Li Q Sun W Ye G Zhang X. Lncrna-rmrp promotes carcinogenesis by acting as a mir-206 sponge and is used as a novel biomarker for gastric cancer. Oncotarget. (2016) 7:37812–24. doi: 10.18632/oncotarget.9336 PMC5122351 27192121

[37] Yue K Ma JL Jiang T Yue J Sun SK Shen JL. Lncrna rpph1 predicts poor prognosis and regulates cell proliferation and migration by repressing P21 expression in gastric cancer. Eur Rev Med Pharmacol Sci. (2020) 24:11072–80. doi: 10.26355/eurrev_202011_23593 33215423

[38] Mi Y Li Y He Z Chen D Hong Q You J. Upregulation of linc-ror promotes the proliferation, migration, and invasion of gastric cancer cells through mir-212-3p/fgf7 axis. Cancer Manag Res. (2021) 13:899–912. doi: 10.2147/CMAR.S287775 33564265 PMC7867499

[39] Ding XZ Zhang SQ Deng XL Qiang JH. Serum exosomal lncrna dlx6-as1 is a promising biomarker for prognosis prediction of cervical cancer. Technol Cancer Res Treat. (2021) 20:1533033821990060. doi: 10.1177/1533033821990060 33550924 PMC7876577

[40] Fawzy MS Toraih EA El-Wazir A Hosny MM Badran DI El Kelish A. Long intergenic non-coding rna, regulator of reprogramming (Linc-ror) over-expression predicts poor prognosis in renal cell carcinoma. Arch Med Sci. (2021) 17:1016–27. doi: 10.5114/aoms.2019.85201 PMC8314397 34336029

[41] Dong P Xiong Y Yue J JBH S Kobayashi N Todo Y. Exploring lncrna-mediated regulatory networks in endometrial cancer cells and the tumor microenvironment: advances and challenges. Cancers (Basel). (2019) 11:234. doi: 10.3390/cancers11020234 30781521 PMC6406952

[42] Zhan HX Wang Y Li C Xu JW Zhou B Zhu JK. Lincrna-ror promotes invasion, metastasis and tumor growth in pancreatic cancer through activating zeb1 pathway. Cancer Lett. (2016) 374:261–71. doi: 10.1016/j.canlet.2016.02.018 26898939

[43] Lu X Chen L Liu S Cao Y Huang Z. M(6)a-mediated upregulation of lncrna rmrp boosts the progression of bladder cancer via epigenetically suppressing scara5. Epigenomics. (2023) 15:401–15. doi: 10.2217/epi-2023-0062 37337726

[44] Li C Zou X Cai Q Li J Yang S Zhang A. Comprehensive expression profile analysis of neutrophil extracellular trap-affected genes in gastric cancer cells and the clinical significance of lncrna neat1-related signaling. Front Oncol. (2022) 12:798531. doi: 10.3389/fonc.2022.798531 35664777 PMC9160368

[45] Chen J Gao Y Zhong J Wu X Leng Z Liu M. Lnc-H19-derived protein shapes the immunosuppressive microenvironment of glioblastoma. Cell Rep Med. (2024) 5:101806. doi: 10.1016/j.xcrm.2024.101806 39481387 PMC11604490

[46] Cupp MA Cariolou M Tzoulaki I Aune D Evangelou E Berlanga-Taylor AJ. Neutrophil to lymphocyte ratio and cancer prognosis: an umbrella review of systematic reviews and meta-analyses of observational studies. BMC Med. (2020) 18:360. doi: 10.1186/s12916-020-01817-1 33213430 PMC7678319

[47] Chen X Liu Q Chen J Liu Y. Lncrna rp11-248e9.5 and rp11-456d7.1 are valuable for the diagnosis of childhood pneumonia. Int J Gen Med. (2021) 14:895–902. doi: 10.2147/IJGM.S291239 33762841 PMC7982557

